# Associations of Conifer-Infesting Bark Beetles and Fungi in Fennoscandia

**DOI:** 10.3390/insects3010200

**Published:** 2012-02-15

**Authors:** Riikka Linnakoski, Z. Wilhelm de Beer, Pekka Niemelä, Michael J. Wingfield

**Affiliations:** 1 Department of Biology, Section of Biodiversity and Environmental Science, University of Turku, Turku 20014, Finland; E-Mail: pekka.niemela@utu.fi; 2 Faculty of Science and Forestry, School of Forest Sciences, University of Eastern Finland, P.O. Box 111, Joensuu 80101, Finland; 3 Department of Microbiology and Plant Pathology, Forestry and Agricultural Biotechnology Institute (FABI), University of Pretoria, Pretoria 0002, South Africa; E-Mails: wilhelm.debeer@fabi.up.ac.za (Z.W.B.); mike.wingfield@fabi.up.ac.za (M.J.W.)

**Keywords:** bark beetles, blue stain, insect-fungus interactions, ophiostomatoid fungi

## Abstract

Bark beetles (Coleoptera, Scolytinae) have a widespread association with fungi, especially with ophiostomatoid fungi (Ascomycota) that cause blue staining of wood, and in some cases, serious tree diseases. In Fennoscandia, most studies of these fungi have focused on economically important bark beetle species and this is likely to have led to a biased view of the fungal biodiversity in the region. Recently, the associations between fungi and bark beetles in Fennoscandia have been shown to be more diverse than previously thought. Furthermore, they form complex and dynamic associations that are only now beginning to emerge. This review examines the current knowledge of the rather poorly known interactions between bark beetles, fungi and their conifer host trees in Fennoscandia. The diversity of ophiostomatoid species is discussed and the possible factors that influence the assemblages of fungal associates are considered for all species that are known to occur in the region. For many ophiostomatoid species found in Fennoscandia, little or nothing is known regarding their pathogenicity, particularly if they were to be transferred to new environments. We, therefore, draw attention to the possible threats of timber trade and climate change-induced invasions of new habitats by bark beetles and the fungi that can be moved along with them.

## 1. Introduction

Forests are important to the welfare of Fennoscandian countries (Fennoscandia: a geographic region which includes the Scandinavian Peninsula, the Kola Peninsula, Karelia and Finland), particularly Finland and Sweden. It is for this reason that the diversity of forest pest species in Fennoscandia is well known [1,2,3,4,5]. In this regard, bark beetles (Coleoptera: Scolytinae) are amongst the most intensively studied forest insects. They include several economically important species, which can cause significant losses to forests and forestry.

With over 6,000 species worldwide, bark beetles are common and geographically widely distributed insects [6]. Lekander *et al*. [2] reported the occurrence of 86 bark beetle species from Fennoscandia and Denmark. These insects occur on a wide range of host trees, including the commercially most important conifer tree species in the boreal forests, Norway spruce (*Picea abies*) and Scots pine (*Pinus sylvestris*). Although some bark beetles species, such as *Ips typographus*, are regarded as important forest pests, the majority of the species is harmless to healthy living trees and infests mainly dead or weakened trees.

Trade in timber with bark or untreated wood has raised the risk of introducing pest species into new environments. Bark beetles are amongst insects that can most easily be moved across national boundaries in raw timber, and they are also well adapted to becoming easily established in new environments [7,8,9,10,11,12]. In addition, bark beetles are associated with symbiotic fungi that substantially complicate the risks of new invasions.

**Figure 1:**
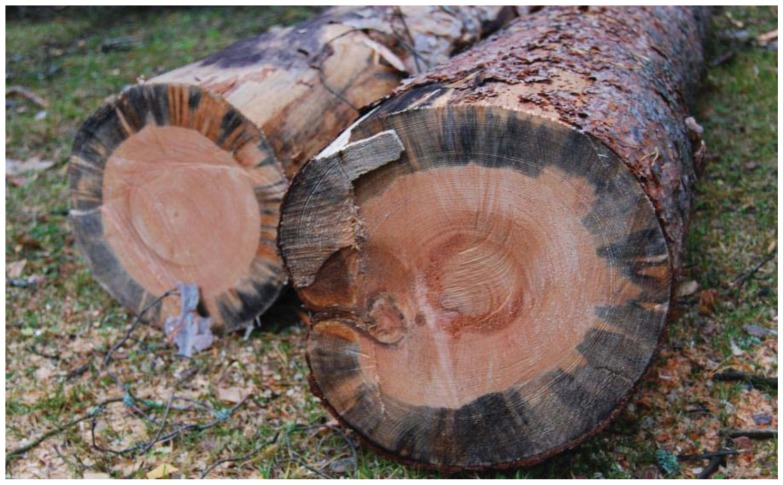
Blue stain caused by ophiostomatoid fungi in *Pinus sylvestris*.

**Figure 2:**
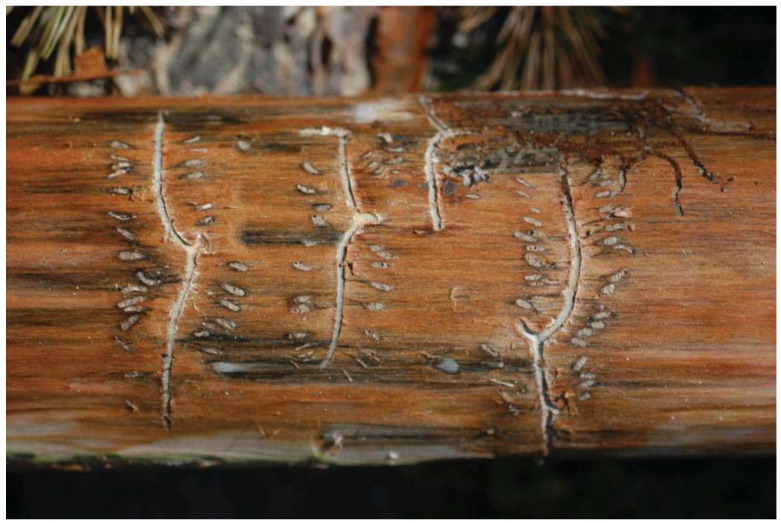
Blue stain caused by ophiostomatoid fungi in the galleries of *Tomicus minor* on *Pinus sylvestris*.

The widespread association between bark beetles and fungi is one of the most fascinating examples of symbioses in nature. The most notable examples are the associations with ophiostomatoid fungi (Ascomycota). Ophiostomatoid fungi mainly cause discoloration of wood, but some species are serious tree pathogens [13,14] (Figure 1 and Figure 2). The associations between bark beetles and ophiostomatoid fungi have probably been shaped during long periods of time. The connection between insect damage and the discoloration of wood was first recognized in the 19th century [15,16,17]. Since the first description of a bark beetle facilitated dispersion of ophiostomatoid fungi, many studies have been devoted to a better understanding of these interactions. In the 20th century, these associations became widely known due to the disastrous Dutch elm disease pandemics killing millions of elm (*Ulmus*) trees in Europe and North America [18,19,20,21]. The complexity of the associations and the difficulty involved in identifying the morphologically similar ophiostomatoid species has meant that they remain poorly understood.

A previous review on the topic listed the bark beetle associated fungi in Europe [13]. Since that time, several new studies have been conducted and they are included here. The occurrences of the species often show biogeographical patterns. For example, the distribution of bark beetles has been explained by the climate and the distribution of the host trees, and is demonstrated to have a strong east-west division in Northern Europe [2,22]. Bark beetle-associated fungi are so poorly known that it is impossible to draw similar conclusions for them. Our preliminary assumption was that the distribution of bark beetle-associated fungi might show similar biogeographical distribution patterns to what has been demonstrated with numerous other organisms, and generally follow the distribution of the host tree and the vector insect. We have, therefore, focused this review on habitat and have considered only a single biogeoraphical area. The aim of this review is to draw together and to study current knowledge regarding bark beetle-fungus associations in a uniform (in climate and vegetation) biogeographical area, the boreal forests of Fennoscandia.

## 2. Bark Beetle-Fungus Symbioses

Given the importance of forests in Fennoscandia, it is unfortunate that there have not been many studies of bark beetle-fungus relationships. The first studies conducted in Fennoscandia took place in the early 20th century and focused on fungi that cause sapstain in pulpwood [23,24,25]. Bark beetles were shown to be directly associated with fungi responsible for discoloration of wood early in the 20th century [26], but no studies were conducted on the bark beetle-associated fungi in Fennoscandia until the 1950s. Rennerfelt [27] and Mathiesen-Käärik [28,29,30,31] carried out pioneering work on this topic in Sweden, and Mathiesen-Käärik’s extensive studies form the basis for Fennoscandian research on bark beetle-associated fungi. Mathiesen-Käärik’s studies remain the most comprehensive investigations for the region. The material for Mathiesen-Käärik’s studies was collected over seven years in different parts of Sweden. The studies included 13 pine and spruce-infesting bark beetle species, including both tree-killing as well as non-tree-killing species. Most of these insects and their fungal associates had not previously been studied elsewhere in the world. In addition to the ecological investigations, Mathiesen-Käärik described numerous new ophiostomatoid species found in the region and carried out physiological studies that included both widely distributed and ecologically restricted ophiostomatoid species. Mathiesen-Käärik observed that species of *Ophiostoma* are the most common fungi associated with bark beetles and that some of these associations can be specific [31]. She hypothesized that these constant associations might result from specific conditions and she conducted experiments to determine conditions favorable for fungal growth. Mathiesen-Käärik observed that different ophiostomatoid fungi have very specific nutritional and moisture requirements, as well as development times. She suggested that these requirements are most likely the key factors determining consistent bark beetle-fungus associations.

Craighead [32] was amongst the first to demonstrate that blue stain fungi might play an important role in the death of bark beetle-infested trees. He also suggested that these fungi might be a source of nutrition for the bark beetles, but this observation has received less attention. Since Craighead’s observations, several studies have been devoted to testing the hypothesis that tree-killing bark beetles require aggressive fungal associates that either kill the tree directly by blocking the water conduction [33,34] or overcome host tree defense mechanisms and thus help the bark beetles in the tree-killing process [35]. This hypothesis, recently referred to as the “classic paradigm” [36], has formed the basis for the majority of studies conducted in Fennoscandia regarding the bark beetle-fungus interactions. Several studies have focused particularly on *I. typographus*, which has caused large-scale outbreaks and serious economic losses in Europe and parts of Fennoscandia [37]. Therefore, this bark beetle species is considered to be the most destructive pest in coniferous forests in Europe. A number of studies have been devoted to searching for a fungus that could play an important role in the initiation and success of *I. typographus* attacks on Norwegian spruce. The fungal associates of *I. typographus* have been investigated in several studies [38,39,40,41], and the pathogenicity of some of these fungal associates has been studied in artificial inoculation trials [42,43,44]. Some of the ophiostomatoid fungi reported in these studies are capable of killing healthy trees, and these species have been considered to be important associates of the beetle species [43].

It is only recently that the “classic paradigms” as defined by Six and Wingfield [36] has been seriously questioned and alternative hypotheses presented to explain the phytopathogenicity of bark beetle-associated fungi [36]. Despite the studies conducted over the last decades, no conclusive evidence has been found to support the classic paradigm [38,39,40,41]. In spite of this fact, the classic paradigm has often been cited as fact in the literature during the last 20–30 years. The earlier studies, e.g., Craighead [32] and Mathiesen-Käärik [28,29,30,31] also discussed the alternative roles of fungi in the lives of bark beetles, but these have gained much less attention during the most recent decades. Craighead [32] suggested that some bark beetles live in nutritional symbioses with fungi, and Mathiesen-Käärik [28,29,30,31] considered how the nutritional requirements of fungi might affect the bark beetle-fungus interactions and thus result in different types of associations. Subsequent to these earlier studies, the classic paradigm, where it was assumed that bark beetles require fungi to infest trees, has been the foundation of much research on this topic.

Six and Wingfield [36] suggested that the virulence of the fungi is probably only one of several characteristics of bark beetle-associated fungi and that these might not have an important role in the ecology of bark beetles. Past research has focused mainly on the possible benefits that fungi might have in the lives of bark beetles. Based on current knowledge, it is obvious that fungi play diverse roles in the ecology of bark beetles and it is important to recognize that not all bark beetle-fungus symbioses are similar. In many cases, these fungi might be considered “weeds” that have developed structures for facilitated insect dispersal without having obvious advantage to the vectoring insect. Virulence might be an important characteristic of fungi, e.g., in mediating competitive interactions with other tree-infesting fungi and supporting their survival in living trees that have active defense mechanisms against fungal invaders [36].

Fungi vectored by non-aggressive bark beetle species have attracted less attention, as the majority of these are actually non-tree-killing species. One of the assumptions in the classic paradigm [36] relating to bark beetle-fungus interactions is that non-aggressive bark beetles would vector fungi that are non-pathogenic or that have low levels of pathogenicity. Only few studies have investigated the fungi associated with non-tree-killing bark beetles in Fennoscandia [28,29,30,31,45,46,47,48]. These studies have shown that both tree-killing and non-tree-killing bark beetles vector relatively similar assemblages of fungi. This raises concern regarding the potential threats that non-aggressive bark beetle species pose, especially if accidentally introduced to new, non-native environments. In this regard, it is well-documented that several fungi vectored by bark beetles are capable of killing trees [39,42,43,44,49], and that invasive strains of fungi, non-pathogenic in their native range, have a potential to cause serious problems in new environments [50,51].

## 3. Ophiostomatoid Fungi Associated with Conifer-Infesting Bark Beetles

Ophiostomatoid fungi are considered the economically most important fungi associated with bark beetles. The morphological features of ophiostomatoid fungi are apparently related to an adaptation to insect dispersal. Despite strikingly similar morphological characteristics and similar niches, different ophiostomatoid genera are phylogenetically unrelated [52]. The fact that some bark beetles have mycangia also supports the view that some associations between bark beetles and ophiostomatoid fungi are highly adapted and evolved during long periods of co-evolution. The economic importance of ophiostomatoid fungi is also important, as many of these fungi are causal agents of serious tree diseases [50,51,53,54,55]. Understandably, bark beetles that vector primary pathogenic fungi are a major concern and studies are commonly focused on describing these associations. However, ophiostomatoid fungi are not the only fungi found with bark beetles. Recent studies have suggested that the ophiostomatoid fungi might not be the dominant species in the overall bark beetle-associated mycobiota [41,56,57]. The focus of several studies has been solely on ophiostomatoid fungi. Therefore, the other possible fungi found together with bark beetles have gained less attention.

Amongst the bark beetle-associated mycobiota, ophiostomatoid fungi remain the most widely studied. However, studies in Fennoscandia have indicated that even more common species have not yet been discovered [46,47,58]. Currently, only a small number of bark beetle species native to the region have been investigated (Table 1), and some of the habitats are also poorly studied. The most common conifer-associated species occurring in Fennoscandia and other parts of Europe reside in the genera, *Ophiostoma *and *Grosmannia* [13,47]. Only one *Ceratocystiopsis*, three *Ceratocystis*, four *Hyalorhinocladiella* [59], and two *Graphium* species have been reported from Fennoscandia. Ophiostomatoid species associated with conifer-infesting bark beetles previously reported from Fennoscandia and remarks on their ecology, distribution and information on their pathogenic properties (when available) are listed below.

**Table 1:** Ophiostomatoid fungi associated with conifer-infesting bark beetles in Fennoscandia.

Bark Beetle	Host Tree	Fungi	References
*Dryocoetes autographus*	*Picea abies*, *Pinus sylvestris*	*Grosmannia cucullata*, *G. olivacea*, *Leptographium chlamydatum*, *L. curvisporum*, *L. lundbergii*, *L. taigensis* nom. prov., *L. truncatum*, *Ophiostoma ainoae*, *O. minus*, *O. piceae*	[46,47,58]
*Hylastes ater*	*P. sylvestris*	*Graphium aureum*, *G. penicillata*, *L. lundbergii*, *L. serpens*, *O. floccosum*, *O. ips*, *O. minus*, *O. piceae*, *O. piliferum*	[28,30]
*H. brunneus*	*P. abies*, *P. sylvestris*	*G. cucullata*, *G. galeiformis*, *L. chlamydatum*, *L. lundbergii*, *O. canum*, *O. pallidulum*, *O. piceae*, *O. tapionis*	[46,47]
*H. cunicularius*	*P. abies*	*G. penicillata*, *G. olivacea*, *G. galeiformis*, *L. chlamydatum*, *L. curvisporum*, *O. piceae *	[30,58]
*Hylurgops palliates*	*P. abies*, *P. sylvestris*	*Ceratocystiopsis minuta*, *Ceratocystis polonica*, *Gr. pycnocephalum*, *G. cucullata*, *G. galeiformis*, *G. penicillata*, *G. piceiperda*, *L. guttulatum*, *L. lundbergii*, *L. procerum*, *L. taigensis* nom. prov., *O. ainoae*, *O. canum*, *O. floccosum*, *O. minus*, *O. piceae*, *O. tapionis*	[28,30,45,46,47,48]
*Ips acuminatus*	*P. sylvestris*	*Cop. minuta*, *C. coerulescens*, *Gr. pycnocephalum*, *Hyalorhinocladiella macrospora*, *L. lundbergii*, *O. canum*, *O. clavatum*, *O. floccosum*, *O. ips*, *O. minus*, *O. piceae*, *O. piliferum*	[27,28,29,30]
*I. amitinus*	*P. abies*	*Cop. minuta*, *O. bicolor*, *O. piceae*	[48]
*I. duplicatus*	*P. abies*	*C. polonica*, *G. penicillata*, *G. piceiperda*, *O. bicolor*, *O. piceae*	[45]
*I. sexdentatus*	*P. sylvestris*	*G. olivacea*, *H. ips*, *H. tingens*, *O. clavatum*, *O. brunneo-ciliatum*, *O. floccosum*, *O. minus*, *O. piceae*, *Pesotum fragrans *	[30,46,47]
*I. typographus*	*P. abies*	*Cop. minuta*, *C. norvegica*, *C. polonica*, *Gr. fimbriisporum*, *Gr. pseudormiticum*, *Gr. pycnocephalum*, *G. cucullata*, *G. olivacea*, *G. penicillata*, *G. piceiperda*, *L. chlamydatum*, *L. taigensis *nom. prov., *O. ainoae*, *O. bicolor*, *O. brunneo-ciliatum*, *O. canum*, *O. flexuosum*, *O. floccosum*, *O. fuscum*, *O. karelicum*, *O. minus*, *O. piceae*, *O. piliferum*, *O. pluriannulatum*, *O. saponiodorum*, *O. stenoceras*, *O. tapionis*, *O. tetropii*, *P. fragrans*	[27,28,29,30,38,40,41,45,46,47,48,60,61,62,63,64,65,66]
*Orthotomicus proximus*	*P. sylvestris*	*C. coerulescens*, *Gr. pycnocephalum*, *L. lundbergii*, *O. clavatum*, *O. floccosum*, *O. ips*, *O. minus*, *O. piceae*, *O. piliferum*, *P. fragrans*	[28,30]
*O. suturalis*	*P. sylvestris*	*G. cucullata*	[47]
*Pityogenes chalcographus*	*P. abies*, *P. sylvestris*	*Cop. minuta*, *C. coerulescens*, *C. polonica*, *Gr. pycnocephalum*, *G. penicillata*, *G. piceiperda*, *L. chlamydatum*, *L. lundbergii*, *L. taigensis *nom. prov., *O. bicolor*, *O. brunneo-ciliatum*, *O. canum*, *O. floccosum*, *O. fuscum*, *O. minus*, *O. piceae*, *O. saponiodorum*, *O. tapionis*, *O. tetropii*	[28,29,30,46,47]
*P. quadridens*	*P. sylvestris*	*G. penicillata*, *H. tingens*, *L. lundbergii*, *O. canum*, *O. minus*, *O. piceae*	[28,30]
*Polygraphus poligraphus*	*P. abies*	*Cop. minuta*, *C. polonica*, *G. penicillata*, *G. piceiperda*, *O. bicolor*	[45]
*Tomicus minor*	*P. sylvestris*	*Cop. minuta*, *G. cucullata*, *H. tingens*, *L. lundbergii*, *O. ainoae*, *O. brunneo-ciliatum*, *O. canum*, *O. floccosum*, *O. karelicum*, *O. minus*, *O. piceae*, *O. piliferum*, *O. pluriannulatum*	[27,28,29,30,46,47]
*T. piniperda*	*P. sylvestris*	*Cop. minuta*, *Gr. pseudormiticum*, *G. cucullata*, *G. olivacea*, *G. penicillata*, *H. tingens*, *L. chlamydatum*, *L. lundbergii*, *L. wingfieldii*, *O. brunneo-ciliatum*, *O. canum*, *O. clavatum*, *O. floccosum*, *O. ips*, *O. minus*, *O. piceae*, *O. piliferum*	[27,28,30,46,47,48,57,67]
*Trypodendron* *lineatum*	*P. abies*, *P. sylvestris*	*Ambrosiella ferruginea*, *G. cucullata*, *G. galeiformis*, *G. penicillata*, *O. canum*, *O. minus*, *O. piceae*, *O. rachisporum*	[30,46,47,48]

### 3.1. Ophiostomatoid Fungi Isolated from Bark Beetles in Fennoscandia: Microascales

#### 3.1.1. *Ambrosiella ferruginea* L.R. Batra 1967

Occurrence: Finland, Sweden.

Only two studies have reported this species in Fennoscandia [30,48]. The fungus has been found in association with *Trypodendron lineatum*. Recent studies confirmed the placement of the species in the Microascales together with four other *Ambrosiella* species [59]. Other species of the *Ambrosiella* previously reported from Fennoscandia have been shown to belong in the Ophiostomatales, and are presently treated in *Hyalorhinocladiella* [59].

#### 3.1.2. *Ceratocystis coerulescens* (Münch) Nannf. 1919

Occurrence: Sweden.

Only one study has reported this species in Fennoscandia. Mathiesen-Käärik [30] found it occasionally from galleries of *Ips acuminatus*, *Orthotomicus proximus* and *Pityogenes chalcographus*. The species can be regarded as an incidental bark beetle associate or a species whose current occurrence in Fennoscandia remains uncertain.

#### 3.1.3. *Ceratocystis norvegica* J. Reid & Hausner 2010

Occurrence: Norway.

A recently described species found in association with *I. typographus* on spruce [60]. The morphological characteristics of the species are similar to other *Ceratocystis* species found on conifers. It is possible that its ecological and morphological similarity to *C. polonica* has hindered the detection of this species in several previous studies. The wider occurrence of the species in the region is currently unknown. An interesting characteristic of the fungus is that it has been shown to be phylogenetically distinct from all other conifer-infesting *Ceratocystis* species that are currently known [60].

#### 3.1.4. *Ceratocystis polonica* (Siemaszko)C. Moreau 1952

Occurrence: Finland, Norway, Sweden, Russian Karelia.

Found only in association with *I. typographus *in Fennoscandia [38,40,41,45,47,61,62,63]. The frequency of the fungus varies greatly at different locations. The species is common in Norway, but rarely found in Finland, Sweden and European parts of Russia. It has been suggested to be essential for *I. typographus* outbreaks, but the results from different studies are conflicting and this view is not currently supported. *Ceratocystis polonica* is considered the most aggressive ophiostomatoid fungus found in Fennoscandia [43,44,68].

#### 3.1.5. *Graphium fimbriisporum* (M. Morelet) K. Jacobs, Kirisits & M.J. Wingf. 2006

Occurrence: Russian Karelia.

Several studies have reported the occurrence of the fungus in Europe in association with spruce-infesting bark beetles [13]. A recent study reported that the species also exists in Fennoscandia [47]. Due to a similar ecology and morphology with synnematous *Ophiostoma *species, *Graphium* species might have been overlooked in previous studies that have based the identification on morphology [69]. These fungi are also sensitive to cycloheximide and they would not have been detected where the antibiotic had been used for isolation purposes.

#### 3.1.6. *Graphium pseudormiticum* M. Mouton & M.J. Wingf. 1994

Occurrence: Sweden, Russian Karelia.

Although it was first described from South Africa [70], this species is considered to have a European origin, but previous reports of its existence in Europe are limited [13,71]. Recent molecular studies have reported the occurrence of the species in Fennoscandia in association with pine and spruce-infesting bark beetles [41,47]. The species might be a more common associate of various conifer-infesting bark beetles than previously believed.

### 3.2. Ophiostomatoid Fungi Isolated from Bark Beetles in Fennoscandia: Ophiostomatales

#### 3.2.1. *Ceratocystiopsis minuta* (Siemaszko) H.P. Upadhyay & W.B. Kendr. 1975

Occurrence: Finland, Sweden, Russian Karelia.

Previous studies reported the occurrence of the fungus in association with pine and spruce-infesting bark beetles in parts of Fennoscandia [30,48,64]. The fungus seems to be a casual associate of several bark beetles. It can be found occasionally in low numbers. The species is a taxonomic challenge and might represent a species complex [72].

#### 3.2.2. *Grosmannia cucullata* (H. Solheim) Zipfel, Z.W. de Beer & M.J. Wingf. 2006

Occurrence: Finland, Norway, Russian Karelia.

The species is commonly found in association with *I. typographus* [38,47,64]. It has also been recently found in association with numerous other bark beetles in Fennoscandia [13,47], as well as in other parts of Europe [13,47]. The species can be regarded as one of the most common *Grosmannia* species found in Fennoscandia. A previous study has revealed that the species might represent a species complex [47].

#### 3.2.3. *Grosmannia galeiformis* (B.K. Bakshi) Zipfel, Z.W. de Beer & M.J. Wingf. 2006

Occurrence: Finland, Sweden, Russian Karelia.

Most collections of the species in Fennoscandia as well as in other parts of Europe were made a relatively long time ago [30,47,73], but the fungus has recently been detected again in the region [47]. Mathiesen-Käärik [30] isolated the fungus from *Hylastes cunicularius*, and a recent study found it associated with *Hylastes brunneus* and two other bark beetle species, *Hylurgops palliatus* and *T. lineatum *[47]. The fungus appears to be a casual associate of mainly secondary bark beetles and can be found occasionally in low numbers.

#### 3.2.4. *Grosmannia olivacea* (Math.-Käärik) Zipfel, Z.W. de Beer & M.J. Wingf. 2006

Occurrence: Finland, Sweden, Russian Karelia.

A relatively common fungus and an example of a species that is found in association with both tree-killing and non-tree-killing bark beetles, such as *Dryocoetes autographus*, *H. cunicularius*, *I. sexdentatus* and *I. typographus* [30,47]. The fungus produces a blue-green stain in timber but is a slow-growing and non-aggressive species [29].

#### 3.2.5. *Grosmannia penicillata* (Grosmann) Goid. 1936

Occurrence: Finland, Norway, Sweden

One of the most common species occurring together with bark beetles and reported in several previous studies [27,30,38,40,41,45,61,62,63]. The fungus is found in association with both tree-killing and non-tree-killing bark beetles, most specifically with *I. typographus*. It has been reported mainly from spruce, but occasionally also from pine. Surprisingly, the most recent studies conducted in Finland and Russian Karelia have not detected the species [47,57,64]. The fungus has a low level of aggressiveness [39].

#### 3.2.6. *Grosmannia piceiperda* (Rumbold) Goid. 1936, = *Grosmannia europhioides* (E.F. Wright & Cain) Zipfel, Z.W. de Beer & M.J. Wingf. 2006

Occurrence: Finland, Norway, Sweden, Russian Karelia.

The taxonomic status of the species is currently unclear. Several studies conducted in Fennoscandia have reported the occurrence of either *G. piceiperda* or *G. europhioides* [38,40,41,45,47,62,64]. Most reports have found the species in association with *I. typographus* on spruce. Some studies have treated the two species as synonyms [74,75,76], but recent phylogenetic studies have indicated that *G. piceiperda* and *G. europhioides* represent distinct species [47,52,77,78]. The existence of these two species in Fennoscandia needs re-evaluation with cultures obtained during previous investigations. Until more data becomes available, we consider *G. piceiperda* and *G. europhioides* as synonyms.

#### 3.2.7. *Hyalorhinocladiella ips* (J.G. Leach, L.W. Orr & C.M. Chr.) T.C. Harr. 2010

Occurrence: Sweden.

Only one previous study reported this species in Fennoscandia [30]. The fungus has been found in association with *Ips sexdentatus* on pine.

#### 3.2.8. *Hyalorhinocladiella macrospora* (Francke-Grosm.) T.C. Harr. 2010

Occurrence: Sweden.

This species has been found in association with *I. acuminatus* on pine in Fennoscandia [30].

#### 3.2.9. *Hyalorhinocladiella tingens* (Lagerb. & Melin) T.C. Harr. 2010

Occurrence: Sweden.

Earlier studies have reported the fungus as a common associate of several pine-infesting bark beetles, including *I. sexdentatus*, *Pityogenes quadridens*, *Tomicus minor* and *Tomicus piniperda*. It has been found most commonly in association with *T. minor* in Sweden [27,28,30]. However, *H. tingens* has not been found during a recent study conducted in other parts of Fennoscandia that included *T. minor* [47].

#### 3.2.10. *Leptographium chlamydatum* K. Jacobs, M.J. Wingf. & H. Solheim 2010

Occurrence: Finland, Norway, Russian Karelia.

The fungus has recently been found in association with root-feeding bark beetles in Norway [58]. The species has also been recently reported from other parts of Fennoscandia [47], and it appears to be a relatively common fungus mainly on spruce in association with several bark beetle species.

#### 3.2.11. *Leptographium curvisporum* K. Jacobs, M.J. Wingf. & H. Solheim 2010

Occurrence: Norway.

A recently described species found in association with root-feeding bark beetles, including *D. autographus* and *H. cunicularius* on spruce [58].

#### 3.2.12. *Leptographium guttulatum* M.J. Wingf. & K. Jacobs 2001

Occurrence: Sweden.

Mathiesen [28] described the fungus *Ophiostoma penicillatum* f. *palliati *from Sweden, and it was later isolated regularly in Europe and described as *L. guttulatum *[79]. The species has been found together with several bark beetles infesting spruce and pine in other parts of Europe. Recent studies, which include relative large surveys in Fennoscandia, have not detected this species.

#### 3.2.13. *Leptographium lundbergii* Lagerb. & Melin 1927

Occurrence: Finland, Sweden, Russian Karelia.

Studies by Mathiesen [28] and Mathiesen-Käärik [30] found *L. lundbergii* (the type species of *Leptographium*) in association with several bark beetle species. Recently, the species re-emerged as a rather common associate of various bark beetle species [47]. It appears to be a common associate of pine-infesting, and mainly secondary, bark beetles. No investigations have found it in association with *I. typographus* and it has received very little attention during the last decades. The fungus is known as an agent of blue stain [80,81] and is weakly pathogenic [82].

#### 3.2.14. *Leptographium taigensis* nom. prov.

Occurrence: Russian Karelia.

A recently found species that apparently occurs as a rather rare, casual associate of several conifer-infesting bark beetles [47]. Its wider geographical distribution and host range is currently unknown.

#### 3.2.15. *Leptographium truncatum* (M.J. Wingf. & Marasas) M.J. Wingf. 1985

Occurrence: Finland.

This fungus was recently detected for the first time from Fennoscandia [47]. It was found in association with pine-infesting *D. autographus*, indicating an association with secondary, stump- and root-colonizing, bark beetle species that have been currently poorly investigated.

#### 3.2.16. *Leptographium wingfieldii* M. Morelet 1988

Occurrence: Norway.

This highly pathogenic fungus has been reported only from Norway in low frequencies together with *T. piniperda* [67,83]. A recent study investigated a large number of *T. piniperda* individuals collected from North and South Finland, but did not detect *L. wingfieldii* [57]. The fungus is a common associate of *T. piniperda* in other parts of Europe, but apparently only a rare species or totally lacking in the northern parts of Europe [13]. *Tomicus piniperda* and *L. wingfieldii* have been recently introduced to North America, where both the insect and the fungus are well established as infesting new host tree species [84].

#### 3.2.17. *Ophiostoma ainoae* H. Solheim 1986

Occurrence: Norway, Russian Karelia.

The fungus is typically found in association with *I. typographus* on spruce, and it can be regarded as a rather common associate of the beetle species [38,40,46,62,63]. However, not all studies have detected the fungus, and its frequency in different locations is variable. The species has also been found occasionally from other pine and spruce-infesting bark beetles [38,40,46,62,63].

#### 3.2.18. *Ophiostoma bicolor* R.W. Davidson & D.E. Wells 1955

Occurrence: Finland, Norway, Sweden, Russian Karelia.

*Ophiostoma bicolor* is one of the most common species found in association with *I. typographus* on spruce [38,41,45,46,48,61,62,64]. It has been only rarely detected from other bark beetles that infest mainly spruce [45,48]. Therefore, the fungus can be regarded as a specific associate of *I. typographus* and it has a low level of aggressiveness [39,44,85].

#### 3.2.19. *Ophiostoma brunneo-ciliatum* Math.-Käärik 1953

Occurrence: Sweden, Russian Karelia.

Only two studies have reported this species in Fennoscandia. *Ophiostoma brunneo-ciliatum* was found first and described by Mathiesen-Käärik [30] from *I. sexdentatus*, and it was recently recorded from Russian Karelia mainly in association with *I. typographus* [47]. The fungus seems to occur as an infrequent associate of mainly *Ips *species in Fennoscandia.

#### 3.2.20. *Ophiostoma canum* (Münch) Syd. & P. Syd. 1919

Occurrence: Finland, Sweden, Russian Karelia.

A common species found mainly in association with pine-infesting bark beetles, such as *T. minor* and *T. piniperda *[27,28,29,30,46,57]. Interestingly, the species has been not reported to occur in Norway [80]. *Ophiostoma canum* is a slow growing fungus with low levels of aggressiveness [83].

#### 3.2.21. *Ophiostoma clavatum* Math.-Käärik 1951

Occurrence: Sweden.

Earlier studies have reported this species in the northern parts of Sweden [27,28,29,30]. The fungus has been found only in association with pine-infesting bark beetles. Most recent studies have failed to detect the species and its occurrence in Fennoscandia is uncertain.

#### 3.2.22. *Ophiostoma flexuosum* H. Solheim 1986

Occurrence: Sweden.

This fungus has been reported in only one study in Fennoscandia. Solheim [38] described the species from galleries of *I. typographus* on spruce. Based on current knowledge, it should be regarded as a rare, incidental bark beetle associate of uncertain distribution in Fennoscandia.

#### 3.2.23. *Ophiostoma floccosum* Math.-Käärik 1951

Occurrence: Sweden, Russian Karelia.

The species is found occasionally on various pine and spruce-infesting bark beetles and their galleries on wood [28,29,30,46]. Its morphological similarity to *Ophiostoma piceae* might have resulted in a failure to detect it in previous studies [86]. The fungus causes blue stain, is known to have a wide distribution [10,86,87] and is not considered pathogenic.

#### 3.2.24. *Ophiostoma fuscum* Linnakoski, Z.W. de Beer & M.J. Wingf. 2010

Occurrence: Finland, Russian Karelia.

A recently described species found in low frequency in association with *I. typographus* and *P. chalcographus* on spruce [46]. The fungus is apparently only an infrequent associate of conifer-infesting bark beetles and its wider distribution is not known.

#### 3.2.25. *Ophiostoma ips* (Rumbold) Nannf. 1934

Occurrence: Sweden.

A study by Mathiesen-Käärik [30] detected the species in low numbers mainly in association with *O. proximus* and other pine-infesting bark beetles in the northern parts of Sweden. Other studies have not found this fungus in Fennoscandia. The fungal associates of pine-infesting secondary bark beetles are poorly investigated and this might suggest that this fungus has been underestimated in the region. *O. ips* has been shown to have low levels of aggressiveness [81].

#### 3.2.26. *Ophiostoma karelicum* Linnakoski, Z.W. de Beer & M.J. Wingf. 2008

Occurrence: Finland, Norway, Russian Karelia.

This fungus is a hardwood-infesting species closely related to the Dutch elm disease fungi [88,89]. It is a consistent associate of the birch bark beetle *Scolytus ratzeburgi*. However, conifer-infesting bark beetles such as *I. typographus* and *T. minor* can also occasionally act as vectors [46].

#### 3.2.27. *Ophiostoma minus* (Hedgc.) Syd. & P. Syd. 1919

Occurrence: Finland, Norway, Sweden, Russian Karelia.

The fungus is one of the most common species, typically found in association with pine-infesting *Tomicus* species. [27,28,30,46,57,67]. It is also associated with several other bark beetle species infesting pine and spruce. *Ophiostoma minus* is considered a relatively aggressive species [67,83,90]. Currently, the taxonomic status of *O. minus* remains unresolved, since European and North American isolate reside in distinct phylogenetic lineages [10,46,91]. European isolates probably correspond to *Ophiostoma pini*, which was originally described from pine in Europe [26], but later considered a synonym of *O. minus *[76].

#### 3.2.28. *Ophiostoma pallidulum* Linnakoski, Z.W. de Beer & M.J. Wingf. 2010

Occurrence: Finland.

A recently described species found in low frequency associated with several different bark beetles [46]. The fungus seems to be an infrequent associate of conifer-infesting bark beetles and its distribution in Fennoscandia is unknown.

#### 3.2.29. *Ophiostoma piceae* (Münch) Syd. & P. Syd. 1919

Occurrence: Finland, Sweden, Norway, Russian Karelia

*Ophiostoma piceae* is probably the most common species found in Fennoscandia [28,30,38,40,41,45,48,62,63,67]. The species is clearly a generalist, occurring in association with a wide range of bark beetles on both pine and spruce. However, a recent survey that applied DNA sequence comparisons for the first time in the region failed to detect isolates of *O. piceae.* Another frequently found species morphologically similar to *O. piceae *but phylogenetically closer to *O. canum* emerged from the study. It is likely that this fungus represents the isolates from Fennoscandia previously identified as *O. piceae* based only on morphological characteristics. Thus, the occurrence of *O. piceae* in the region should be considered unconfirmed at the present time. *Ophiostoma piceae* is a non-pathogenic, sapwood staining species [68].

#### 3.2.30. *Ophiostoma piliferum* (Fr.) Syd. & P. Syd. 1919

Occurrence: Finland, Norway, Sweden.

*Ophiostoma piliferum* (the type species of *Ophiostoma*) occurs rarely in association with pine-infesting bark beetles [28,30,48,67]. It has been detected occasionally from *I. typographus* on spruce [48]. The species is considered economically important as a sapstain agent, and also for a white mutant (marketed as Cartapip 97^®^) that is used for pulping and blue stain control [92].

#### 3.2.31. *Ophiostoma pluriannulatum* (Hedgc.) Syd. & P. Syd. 1919

Occurrence: Sweden.

The species is found only occasionally in association with conifer-infesting bark beetles [28,30]. The last reports of the species were made several decades ago, and they might have represented any of the morphologically similar species [93]. Therefore, the current occurrence of the species in Fennoscandia is unclear.

#### 3.2.32. *Ophiostoma rachisporum* Linnakoski, Z.W. de Beer & M.J. Wingf. 2010

Occurrence: Finland, Russian Karelia.

A recently described species found mainly in association with *T. lineatum* [46]. The fungus might be a common associate of the beetle species but its wider distribution in the region is currently unknown.

#### 3.2.33. *Ophiostoma saponiodorum* Linnakoski, Z.W. de Beer & M.J. Wingf. 2010

Occurrence: Finland, Russian Karelia.

A recently described fungus that is one of the most common species associated with *P. chalcographus* [46]. The fungus might be a common associate of the beetle species, but its wider occurrence in Fennoscandia remains unknown.

#### 3.2.34. *Ophiostoma stenoceras* (Robak) Melin & Nannf. 1934

Occurrence: Sweden.

The species is found only occasionally in association with conifer-infesting bark beetles [28,30]. The last reports of the species were many years ago and its current occurrence in Fennoscandia is uncertain.

#### 3.2.35. *Ophiostoma tapionis* Linnakoski, Z.W. de Beer & M.J. Wingf. 2010

Occurrence: Finland, Russian Karelia.

A recently described fungus commonly found in association with *H. palliatus* on pine and spruce, and occasionally from other beetles [46]. The fungus might be a common associate of the beetle species, but its wider occurrence in Fennoscandia remains unknown.

#### 3.2.36. *Ophiostoma tetropii* Math.-Käärik 1951

Occurrence: Finland, Norway, Sweden.

The fungus is a casual associate of mainly *I. typographus* on spruce [38,40,48,63]. Previous studies have detected the species only rarely, and in variable frequencies. In some localities the fungus is frequently found. *Ophiostoma tetropii* is non-aggressive [94].

#### 3.2.37. *Pesotum fragrans* (Math.-Käärik) G. Okada & Seifert 1999

Occurrence: Finland, Sweden, Russian Karelia.

The taxonomic status of the fungus is unresolved. The so-called *P. fragrans* isolates have been occasionally collected from several conifer-infesting bark beetles in Fennoscandia [30,47]. These isolates seem to form a distinct lineage in the Ophiostomatales, possibly representing a new genus [95].

## 4. Other Fungal Associates

The other fungi associated with bark beetles include other ascomycetes, basidiomycetes and zygomycetes [13]. In general, ascomycetes are the most abundant fungi found in association with bark beetles. Several studies have detected a great number of other species belonging to the Ascomycota [40,41,47,56,57]. These fungi include several cosmopolitan saprotrophs and species that are plant or insect pathogens. Their common occurrence is, therefore, not surprising. Yeasts (both basidiomycetes and ascomycetes) are also commonly encountered in association with beetles and their galleries. Their taxonomy is still rather poorly understood, but the majority seem to belong to the Ascomycota [47,56,57,96]. The yeasts found in Fennoscandia include species in the genera *Candida*, *Cryptococcus*, *Kuraishia*, *Pichia *and *Saccharomyces. *Somezygomycetous fungi are considered casual associates, but few species have been reported [41,47,57]. The zygomycetes include chitinolytic fungi such as bark beetle-associated *Mortierella* species, which are involved in the degradation process of chitinous exoskeletons of insects [97].

*Heterobasidion annosum *causesa destructive root rot disease of conifers, and the fungus is particularly important in the boreal forests of Fennoscandia [98]. Its potential association with insects in Fennoscandia has not been investigated until recently [41,56,57]. Harding [85] detected *H. annosum* in association with conifer-infesting bark beetles in Denmark. Studies by Persson *et al*. [41,56] did not find the fungus occurring together with the studied bark beetle species, but could not neglect the bark beetle role as a potential vector of *H. annosum*. The studies found a surprisingly large number of other wood decay basidiomycetes occurring together with *I. typographus*. Most likely, the interactions between bark beetles and basidiomycetes are unspecific, but some species could also be more intimately associated with a certain bark beetle species, as studies in other parts of Europe and North America have revealed [13,96,99].

There is emerging evidence that basidiomycetes are more common associates of bark beetles than previously thought. These fungi have occasionally and probably unintentionally been found in bark beetle studies, since the focus of the studies had been on ophiostomatoid fungi [40,47]. Interestingly, three different studies have reported the occurrence of a white-rot fungus *Bjerkandera adusta* together with *I. typographus* in Finland and Sweden [40,41,56]. Based on the results of recent studies that have applied more accurate identification methods, it is likely that the fungal isolation methods commonly applied in the bark beetle investigations select against basidiomycetes [41,56,57]. Relatively extensive surveys that have applied traditional isolation techniques, have failed to detect the apparently common occurrence of basidiomycetes [30,47,57]. Therefore, the diversity of the basidiomycetes as bark beetle-associates has been most likely underestimated.

## 5. Do Tree-Killing and Non-Tree-Killing Bark Beetles Vector Different Fungi?

The economically most important bark beetle species, *I. typographus*, is by far the most intensively studied bark beetle species in Fennoscandia [28,29,30,38,40,41,46,47,61,62]. The fungal associates of *I. typographus* have been investigated in different locations, and in both endemic and epidemic conditions. Several studies have also reported the fungal associates of other bark beetle species in the region, including both aggressive and non-tree-killing insect species [28,29,30,45,46,47,48,57,58]. Amongst these, the fungal associates of the pine shoot beetle *T. piniperda* might be regarded as relatively well known. Previous studies have recorded the fungal associates of altogether 17 different pine- and spruce-infesting bark beetles in Fennoscandia [47]. The associations between bark beetles and fungi in all of Europe have recently been reviewed by Kirisits [13]. The species studied in Fennoscandia represent only a small proportion of the bark beetle species native to the region [2]. Unlike the extensively studied *I. typographus* and the rather well known *T. piniperda*, the fungal associates of many of the other bark beetle species remain poorly investigated. Only one or perhaps a few studies have focused on a small number of individual bark beetles. It is, therefore, difficult to establish comprehensive conclusions regarding the fungal associates of several bark beetle species, and a broad interrogation of the topic will only be possible when greater numbers of insects and their fungal associates have been considered.

The assemblage of fungi vectored by *I. typographus* is well known, although various aspects of the associations remain unknown. Several ophiostomatoid fungi have been consistently and regularly found associated with *I. typographus* [27,28,29,30,38,40,41,46,47,48,61,62,63,64,48,61]. In most cases, these associations appear to be unspecific. This implies that every individual bark beetle carries spores of several fungi, but in a bark beetle population the majority of these fungal associates are found only occasionally [13]. Therefore, the majority of fungi associated with *I. typographus* could be regarded as casual or occasional associates. Although different studies, and the locations where they have been conducted, show substantial variation, a few fungal species have been reported in the majority of the investigations conducted in the region. These fungi can be regarded as constant associates of *I. typographus*. These include *C. polonica*, *O. ainoae*, *O. bicolor*, *G. penicillata* and *G. piceiperda*.

Studies conducted in Norway on *I. typographus* have shown that the highly aggressive fungus *C. polonica* is frequently associated with the beetle [38,43,62]. Because the fungus was commonly encountered and its aggressiveness demonstrated in several studies [42,43,44,49], *C. polonica* has been suggested as playing a role in killing spruce trees. However, studies conducted in Finland, Sweden and European parts of Russia have detected the species only occasionally [33,40,41,47,56,63,64]. In some locations the species is only rarely detected, while in others the species is considered the most common fungal associate of *I. typographus* [100]. This fact supports the view that *I. typographus* is unlikely to require *C. polonica* to kill trees [36].

It has been documented that the less-economically important (non-tree-killing) bark beetles vector ophiostomatoid fungi in Fennoscandia [28,29,30,45,46,47,48], as well as in the other parts of Europe [13,68,85,99]. Interestingly, the general assemblage of fungi on tree-killing and non-tree-killing bark beetles is similar and both groups carry several ophiostomatoid species. The majority of the associated fungi are found inconsistently and in low numbers, indicating casual associations. A few fungi are found more commonly together with certain beetle species, apparently indicating a more specific association [47]. In addition to the consistent associates of *I. typographus*, rather well known examples include e.g., *O. canum* and *O. minus* associated with *T. minor* and *T. piniperda* [30,47,57,67].

In several cases, the same fungal species are found associated with both tree-killing and non-tree-killing bark beetles. These include e.g., *G. piceiperda*, *L. lundbergii*, *O. minus* and *O. piceae* [47]. One of the precepts of the classic paradigm to explain the role of fungi in the lives of bark beetles is that tree-killing bark beetles are more commonly associated with virulent fungi, and that beetles that do not kill trees either lack fungal associates or they are non-pathogenic [36]. Based on the current knowledge, it is clear that the non-tree-killing bark beetles are also commonly associated with ophiostomatoid fungi [28,29,30,45,46,47,48,68]. For example, *O. minus *and *G. piceiperda* are fungi considered to be relatively pathogenic and they are found in association with non-tree-killing bark beetles [30,46]. Only a few fungi appear to be restricted to a single bark beetle species. In Fennoscandia, the best-known example is *C. polonica* associated with *I. typographus.* In contrast, the majority of the fungi are typically either rare bark beetle associates in nature or they are known from very small numbers of investigations or investigations that have been conducted in poorly studied niches.

If fungal aggressiveness were a requirement in the tree-killing process, evolutionary selection pressure would lead to highly consistent interactions between bark beetles and fungi [36]. Such associations between conifer-infesting beetles and fungi have not been described. However, current knowledge cannot confirm this argument against the classic paradigm. The occurrence of the most pathogenic species in Fennoscandia, *C. polonica*, is highly variable. It is important to note that studies in Norway that have found the fungus as a common associate of *I. typographus*, have been conducted in outbreak conditions [38,44,45,61,68], while other Fennoscandian studies represent mainly non-outbreak conditions [40,41,48,63]. Although various other factors could explain the conflicting results emerging from these different studies, it remains possible that other fungi could replace the most virulent species like *C. polonica* during non-outbreak conditions [101]. However, there is no clear evidence to support the suggestion that the fungi associated with bark beetles differ from outbreak to non-outbreak conditions. In addition, the mechanism that would support the potential shifts of the fungi depending upon bark beetle population phase is unknown.

Based on current knowledge, it is recognized that bark beetles vector fungi with different degrees of aggressiveness [40,44,47,68,67]. Six and Wingfield [36] suggested that virulence should be viewed as a fungal character that might have a more important role for the fungus itself than for the bark beetle. Testing this hypothesis could provide new insights into bark beetle-fungi interactions in the future.

## 6. What Causes the Variation in the Assemblages of Fungi?

Some fungal species occur at variable frequencies in different localities and even within the same bark beetle population. Very little is known regarding the factors that influence this variation; the mechanisms are also difficult to investigate. Regional differences, such as geographical variation and differences in forestry practices, might explain part of the observed variation. Other factors that could be important include differences in the sampling methodology, different levels of effort applied to investigations in different regions, the experience and focus of the researchers involved, and the isolation methods used in different studies [13]. These factors will greatly influence the results and complicate direct comparisons between different studies. To add to this complication, it is not known how different biotic and abiotic factors might affect the assemblages of fungi over time [36].

Temperature plays a key role in the relative abundance of some fungal associates. Several studies have shown that ophiostomatoid fungi differ in their growth rate at certain temperatures, and each species has a unique optimal growth temperature [38,46,83]. The different temperature requirements of fungi determine the variety of fungi vectored by a bark beetle at ambient temperatures [102,103]. Six and Bentz [102] suggested that different but overlapping environmental tolerances could result in the coexistence of multiple fungal associates. The importance of temperature has also been shown in recent studies conducted in Fennoscandia. Persson *et al*. [41] demonstrated that a warming climate causes a shift in the overwintering niche of bark beetles from forest litter or fallen trees to standing trees, and also affects the fungal communities associated with the hibernating bark beetles. Fungi associated with specific beetles vary between Northern and Southern Finland, indicating that geographical and climatic differences might play a role in determining the assemblages of fungi [57].

Studies that consider the effect of temperature, latitude or season are few in number [57,102,103]. There is also a lack of attempts to investigate and compare the fungal associates at the same time and in similar environments between outbreak and non-outbreak areas. This information could be important to enable a better understanding of the roles of fungi in the lives of aggressive bark beetles.

Methodological strategies applied by different researchers most likely significantly influence the outcomes of different studies. Sampling conducted in Fennoscandia has included various strategies. In several studies, bark beetles and their galleries have been collected manually from naturally infested trees, from trapping logs that have been laid on the forest floor, and/or from logs and material from saw-mills or timber yards [27,28,30,41,56,57,58,62]. In some studies, bark beetle attacks on trees are induced by pruning living trees or by using pheromone dispensers [38,67]. Bark beetles for fungal isolation have been also collected in pheromone traps [40,62,63]. Different trapping methods have been demonstrated to affect the frequencies of fungi isolated from beetles. A study by Viiri [40] compared individually collected and pheromone trapped bark beetles and demonstrated that these methods could result in differences in the detection of fungi. This could be related to the roles that phoretic mites play in the dispersal of the fungi [104]. Another potential problem with pheromone trapping is that it could result in cross-contamination when the trapped beetles are used for investigating the fungal associates of individual beetles.

A proportion of the fungal assemblage associated with bark beetles could remain undetected due to selective isolation methods that overlook slow-growing or an unculturable fraction of the fungi. In Fennoscandian studies, mainly two different isolation methods have been applied. In a direct isolation method, fungi have been isolated directly, either from lesions on wood, from insect galleries or from beetles at different stages of development [27,28,29,30,38,46,47,58,62,67]. Using this approach, the samples have been placed on malt extract agar, sometimes containing cycloheximide [46,47,58], then the fungi have been allowed to grow and sporulate. During the incubation period, different fungal species have been detected and transferred to new media, eventually resulting in pure fungal cultures. In contrast, using an indirect isolation method, living beetles have been inoculated in sterilized logs and fungi have been isolated from the stained wood [40,45,61,62].

Clearly, whichever method has been used for fungal isolation could have a significant influence on the detection of some species [13]. The direct isolation method seems to select against some species. Species that were difficult to isolate using the direct isolation method include *C. polonica* and *G. penicillata* [61], and it must also be noted that when cycloheximide has been added to the medium, species of *Ceratocystis* and *Ceratocystiopsis *would not grow [105,106]. Studies employing the indirect isolation method reported the common occurrence of *G. penicillata *in association with *I. typographus* [40,45,62,63]. Recent surveys using the direct isolation method did not detect *G. penicillata* [47]. It is also known from studies on other fungi, that the culturable fraction of fungal associates does not represent the whole fungal diversity, because slow-growing fungi and species present at low frequencies are difficult to detect [107].

Identification methods have a significant impact on the detection of different species. Traditionally, the identification of ophiostomatoid fungi has been based on morphological characteristics. Ophiostomatoid fungi inhabit similar niches and have a simple morphology with many overlapping features. This has resulted in decades of confused taxonomy and intense debate dating back to virtually the first descriptions of these fungi. Several recent studies have demonstrated that DNA sequence-based methods in combination with morphological characteristics are essential for precise species identification [47,52,108]. It is already evident that the true diversity of ophiostomatoid species is underestimated and that several common species have not been discovered. This has been demonstrated in a recent survey where several new species from a small geographical area were found [46,47]. The advantage of new molecular techniques is that they allow the direct identification of fungi on beetles, resulting in more accurate estimates of the true fungal biodiversity. Persson *et al*. [41,56] applied a method for analyzing the non-culturable fraction of bark beetle-associated fungi and demonstrated that the detection method has a significant impact on the observed fungal diversity.

## 7. Timber Trade and Climate Change—A Risk?

Bark beetles and their fungal associates are important components of coniferous forest ecosystems. They have an essential role in nutrient cycling and succession dynamics in forest ecosystems [109]. The relationships between bark beetles, fungi and host trees have developed over millions of years of co-evolution, which has resulted in complex and dynamic associations. The killing of trees and occasional outbreaks of several bark beetle species are essential processes in the succession dynamics of natural forest ecosystems. How global climate change will affect these outbreaks remains poorly understood [109].

Human activities such as forest management, introduction of invasive species through global trade, and loss of biodiversity have drastically changed forest ecosystems and disturbed the interactions between different organisms. Consequently, these factors may have strong effects on bark beetle outbreaks [110,111]. Martikainen *et al*. [111] demonstrated that in old forests with a rich biodiversity and a constant supply of dying trees, secondary bark beetles are more abundant and populations of primary bark beetles remain at non-epidemic levels. Several recent examples have also shown that over the past decades, the frequency, severity and extent of bark beetle outbreaks has increased [112].

It was not until the early decades of the 20th century, with the expansion and mechanization of the forestry and forest products industries, that the socio-economic importance of bark beetle-fungi interactions became evident. The most widely known example of a human-induced invasion of bark beetle-associated fungi to new areas and its destructive consequences is found in the Dutch elm disease fungi vectored by *Scolytus*-species. These fungi are native to Asia and investigations have traced the origin of some species to the Himalayas [50,113]. Unlike the Asian elms, elms endemic to Europe and North America lack the resistance to the Dutch elm disease fungi [114,115]. The disease emerged after World War I and spread rapidly in Europe and from there to North America. Its introduction led to devastating effects, killing the majority of the original elm populations. As the Dutch elm disease pandemics have shown, the elimination of the fungal pathogens can be extremely difficult once the disease epidemics begin. Several attempts to control Dutch elm disease have failed, and the disease remains epidemic. Therefore, the most effective means to control fungal diseases such as those listed above is to prevent introduction of the pathogens to new environments.

Increased global trade and travel have increased the rates of forest pest introductions to new environments [116]. Moreover, preventing the introduction of pests and pathogens into new areas is beset with challenges. Several countries, including Finland and Sweden, rely substantially on Russia as a source of raw timber [117]. Possible pest and pathogen risks involved in the timber imported from Russia to other countries are difficult to predict based on current knowledge. For example, there are few studies considering pests and pathogens that might be moved across national boundaries on untreated timber. Siitonen [11] identified several potential pest species not native to Finland on timber originating from Russian Siberia. Recent studies have clearly shown that the available knowledge regarding micro-organisms that are potentially transferred with bark beetles and untreated timber remains rather poor. These investigations have further demonstrated that the fungal diversity associated with bark beetles is much greater than previously thought [41,46,47,56]. Fungal associates of only a few bark beetle species are well-known and even less is known regarding the potential pathogenicity of the majority of bark beetle-associated fungi. Moreover, it is important to recognise that even fungi that are non-pathogenic in their native environment have the potential to cause serious disease problems in new environments.

We know very little about the possible impact of climate change and how this might influence invasions of both native and exotic insects [116]. Climate change could, for example, affect the bark beetle reproduction and population dynamics and increase the frequency and intensity of outbreaks [118,119]. Damage caused by *I. typographus* in Finland has been low compared to that in southern Europe. This has been explained by the fact that *I. typographus* has only one generation a year in the northern parts of its range and more than one generation in other parts of Europe. It is, therefore, worrying that *I. typographus* was recently shown to complete two generations per year for the first time in recorded history in Finland and Sweden [120,121].

Climate change might have direct effects on insect performance and behaviour. Persson *et al*. [41] have, for example, demonstrated that a warming climate could result in a shift in the overwintering niche of bark beetles. How climate change will alter the bark beetle-fungus interactions is largely unknown. It has been demonstrated that temperature can have a significant role in shaping these associations [102,103]. Thus, different temperature requirements of fungi can influence the fungal associates that are vectored by the bark beetle at ambient temperature [102,103]. A change in the insect behavior, such as a shift in the overwintering strategy, can also have significant impact on the fungal associates of the beetles [102].

## 8. Conclusions

The results of recent investigations have shown that the fungal diversity associated with conifer-infesting bark beetles in Fennoscandia is greater than previously recognized [41,46,47,56]. Ophiostomatoid fungi are commonly found in this area and they are the best-known associates of bark beetles. The ophiostomatoid species reported from Fennoscandia apparently represent species typical to the region. However, predicting the risks involved in timber trade and climate change is difficult based on current knowledge.

Information regarding the pathogenicity of ophiostomatoid fungi to endemic host trees or to potentially new host trees is lacking in many cases. Clearly our understanding of the different interactions between non-tree-killing bark beetles and their fungal associates is incomplete. Some of these species are the same as those that can be found in association with tree-killing bark beetles, also including pathogenic fungi. The roles of the fungi in the life histories of bark beetles, and how different biotic and abiotic factors might affect these insect-fungus interactions remains poorly known. We, therefore, subscribe to the view that all bark beetle species and the fungi associated with them should be considered potential threats to forest health. This is especially the case where they are accidentally introduced to new areas where they are not native.

## References

[1] Heliövaara K., Peltonen M., Mannerkoski I., Siitonen J. (1998). Suomen kaarnakuoriaiset (Coleoptera: Scolytidae).

[2] Lekander B., Bejer-Petersen B., Kangas E., Bakke A. (1977). The distribution of bark beetles in the Nordic countries. Acta Entomol. Fenn..

[3] Mandelshtam M.J., Popovitchev B.G. (2000). Annotated check-list of bark-beetles (Coleoptera: Scolytidae) of Leningrad oblast. Entomol. Obozr..

[4] Saalas U. (1949). Suomen metsähyönteiset.

[5] Voolma K. (2004). Distribution and spread of bark beetles (Coleoptera: Scolytidae) around the Gulf of Finland: A comparative study with notes on rare species of Estonia, Finland and North-Western Russia. Entomol. Fenn..

[6] Bright D.E. (1976). The bark beetles of Canada and Alaska. The Insects and Arachnids of Canada, Part. 2, The Bark Beetles of Canada and Alaska (Coleoptera: Scolytidae).

[7] Ericson B. (2010). Två för Sverige nya skalbaggar (Coleoptera) som angriper lärk. Ent. Tidskr..

[8] Gillefors G. (1988). Skalbaggar införda till Sverige med importerad massaved. Ent. Tidskr..

[9] Lundberg S. (1988). Några intressanta skalbaggsfynd i till Norrbotten importerat barrvirke. Ent. Tidskr..

[10] Min L., Zhou X.D., de Beer Z.W., Wingfield M.J., Sun J.-H. (2009). Ophiostomatoid fungi associated with the invasive pine-infesting bark beetle, *Dendroctonus valens*, in China. Fungal Divers..

[11] Siitonen J. (1990). Potential forest pest beetles conveyed to Finland on timber imported from the Soviet Union. Silva Fenn..

[12] Tkacz B.M. (2002). Pest risk associated with importing wood to the United States. Can. J. Plant Pathol..

[13] Kirisits T., Lieutier F., Day K.R., Battisti A., Grégoire J.-C., Evans H. (2004). Fungal associates of European bark beetles with special emphasis on the ophiostomatoid fungi. Bark and Wood Boring Insects in Living Trees in Europe, a Synthesis.

[14] Wingfield M.J., Seifert K.A., Webber J.F. (1993). Ceratocystis and Ophiostoma: Taxonomy, Ecology and Pathogenicity.

[15] Schmidberger J. (1836). Naturgeschichte des Apfelborkenkäfers *Apate dispar*. Beiträge zur Obstbaumzuchtund zur Naturgeschichte der den Obstbäumen schädlichen Insekten.

[16] Hartig T. (1844). Ambrosia des *Bostrichus dispar*. Allgemeine Forst und Jagzeitung.

[17] Hartig R. (1878). Die Zersetzungserscheinungen des Holsez der Nadelbäume und der Eiche in forstlicher, botanischer und chemischer Richtung.

[18] Brasier C.M. (1991). *Ophiostoma novo-ulmi* sp. nov., causative agent of current Dutch elm disease pandemics. Mycopathologia.

[19] Brasier C.M., Kirk S.A. (2001). Designation of the EAN and NAN races of *Ophiostoma novo-ulmi* as subspecies: Their perithecial size differences and geographical distributions. Mycol. Res..

[20] Gibbs J.N. (1978). Intercontinental epidemiology of Dutch elm disease. Annu. Rev. Phytopathol..

[21] Hubbes T. (1999). The American elm and Dutch elm disease. For. Chron..

[22] Heliövaara K., Väisänen R., Immonen A. (1991). Quantitative biogeography of the bark beetles (Coleoptera, Scolytidae) in Northern Europe. Acta For. Fenn..

[23] Wegelius T. (1938). Sulphite wood decay and its influence on the manufacturing process and pulp yield. Finsk Papp. Tidskr..

[24] Lagerberg T., Lundberg G., Melin E. (1927). Biological and practical researches into blueing in pine and spruce. Sven. Skogsvårdsfören. Tidskr..

[25] Nannfeldt J.A., Melin E. (1934). Researches into the blueing of ground-wood pulp. Sven. Skogsvårdför. Tidskr..

[26] Münch E. (1907). Blaufäule des Nadelholzes. I-II. Naturwissenschaftliche Zeitschrift für Forst und Landwirtschaft.

[27] Rennerfelt E. (1950). Über den Zusammenhang zwischen dem Verblauen des Holzes und den Insekten. Oikos.

[28] Mathiesen A. (1950). Über einige mit Borkenkäfern assoziirte Bläuepilze in Schweden. Oikos.

[29] Mathiesen A. (1951). Einige neue *Ophiostoma*-Arten in Schweden. Svensk Bot. Tidskr..

[30] Mathiesen-Käärik A. (1953). Eine Übersicht uber die gewöhnlichsten mit Borkenkäfern assoziierten Bläuepilze in Schweden einige für Sweden neue Bläuepilze. Med. Stat. Skogsf. Inst. Stockholm..

[31] Mathiesen-Käärik A. (1960). Studies on the ecology, taxonomy and physiology of Swedish insect-associated blue stain fungi, especially the genus *Ceratocystis*. Oikos.

[32] Craighead F.C. (1928). Interrelation of tree-killing bark beetles (*Dendroctonus*) and blue stains. J. For..

[33] Långström B., Solheim H., Hellqvist C., Gref R. (1993). Effects of pruning young Scots pine on host vigor and susceptibility to *L. wingfieldii* and *Ophiostoma minus*, two blue stain fungi associated with *Tomicus piniperda*. Eur. J. For. Pathol..

[34] Paine T.D., Raffa K.F., Harrington T.C. (1997). Interactions among scolytid bark beetles, their associated fungi, and live host conifers. Annu. Rev. Entomol..

[35] Lieutier F., Yart A., Salle A. (2009). Stimulation of tree defenses by ophiostomatoid fungi can explain attack success of bark beetles in conifers. Ann. For. Sci..

[36] Six D.L., Wingfield M.J. (2011). The role of phytopathogenicity in bark beetle-fungus symbioses: A challenge to the classic paradigm. Annu. Rev. Entomol..

[37] Christiansen E., Bakke A., Berryman A.A. (1998). The spruce bark beetle of Eurasia. Dynamics of Forest Insect Populations.

[38] Solheim H. (1986). Species of Ophiostomataceae isolated from *Picea abies* infested by the bark beetle *Ips typographus*. Nord. J. Bot..

[39] Horntvedt R., Christiansen E., Solheim H., Wang S. (1983). Artificial inoculation with *Ips typographus*-associated blue-stain fungi can kill healthy Norway spruce trees. Medd. Nor. Inst. Skogforsk..

[40] Viiri H. (1997). Fungal associates of the spruce bark beetle *Ips typographus* L. (Coleoptera: Scolytidae) in relation to different trapping methods. J. Appl. Entomol..

[41] Persson Y., Vasaitis R., Långström B., Öhrn P., Ihrmark K., Stenlid J. (2009). Fungi vectored by the bark beetle* Ips typographus* following hibernation under the bark of standing trees and in the forest litter. Microb. Ecol..

[42] Christiansen E. (1983). *Ceratocystis polonica* inoculated in Norway spruce: Blue-staining in relation to inoculum density, resinosis and tree growth. Eur. J. Forest Pathol..

[43] Christiansen E., Solheim H. (1990). The bark beetle-associated blue stain fungus *Ophiostoma polonicum* can kill various spruces and Douglas fir. Eur. J. Forest Pathol..

[44] Solheim H. (1988). Pathogenicity of some *Ips typographus*-associated blue-stain fungi to Norway spruce. Medd. Nor. Inst. Skogforsk..

[45] Krokene P., Solheim H. (1996). Fungal associates of five bark beetle species colonizing Norway spruce. Can. J. For. Res..

[46] Linnakoski R., de Beer Z.W., Ahtiainen J., Sidorov E., Niemelä P., Pappinen A., Wingfield M.J. (2010). *Ophiostoma* spp. associated with pine- and spruce-infesting bark beetles in Finland and Russia. Persoonia.

[47] Linnakoski R. (2011). Bark Beetle-Associated Fungi in Fennoscandia with Special Emphasis on Species of *Ophiostoma* and *Grosmannia*. Ph.D. Dissertation.

[48] Savonmäki S. (1990). Tärkeimmät kaarnakuoriaisten mäntyyn ja kuuseen levittämät sinistäjäsienilajit. M.Sc. Thesis.

[49] Solheim H., Safranyik L. (1997). Pathogenicity to Sitka spruce of *Ceratocystis rufipenni* and *Leptographium abietinum*, blue stain fungi associated with the spruce bark beetle. Can. J. For. Res..

[50] Brasier C.M. (1983). Dutch elm disease. The Origin of Dutch elm Disease; Report of Forest Research 32.

[51] Lu M., Wingfield M.J., Gillette N.E., Mori S.R., Sun J.-H. (2010). Complex interactions among host pines and fungi vectored by an invasive bark beetle. New Phytol..

[52] Zipfel R.D., de Beer Z.W., Jacobs K., Wingfield B.D., Wingfield M.J. (2006). Multi-gene phylogenies define *Ceratocystiopsis* and *Grosmannia* distinct from *Ophiostoma*. Stud. Mycol..

[53] Harrington T.C., Wingfield M.J., Seifert K.A., Webber J.F. (1993). Diseases on conifers caused by species of *Ophiostoma* and *Leptographium*. Ceratocystis and Ophiostoma: Taxonomy, ecology and pathogenicity.

[54] Kile G.A., Wingfield M.J., Seifert K.A., Webber J.F. (1993). Plant diseases caused by species of *Ceratocystis sensu stricto* and *Chalara*. Ceratocystis and Ophiostoma: Taxonomy, Ecology and Pathogenicity.

[55] Roux J., Wingfield M.J. (2009). *Ceratocystis *species: Emerging pathogens of non-native plantation *Eucalyptus* and *Acacia* species. South. Forests.

[56] Persson Y., Ihrmark K., Stenlid J. (2011). Do bark beetles facilitate the establishment of rot fungi in Norway spruce?. Fungal Ecol..

[57] Silva X. (2011). Population Studies of Fungi Associated with *Tomicus piniperda* L. in Finland. M.Sc. Thesis.

[58] Jacobs K., Solheim H., Wingfield M.J. (2010). Two new species of *Leptographium* from *Dryocetes autographus* and *Hylastes cunicularius* in Norway. Mycol. Prog..

[59] Harrington T.C., Aghayeva D.N., Fraedrich S.W. (2010). New combinations in *Raffaelea*, *Hyalorhinocladiella*, and *Hyalorhinocladiella*, and four new species from the redbay ambrosia beetle, *Xyleborus glabratus*. Mycotaxon.

[60] Reid J., Iranpour M., Rudski S.M., Loewen P.C., Hausner G. (2010). A new conifer-inhabiting species of *Ceratocystis* from Norway. Botany.

[61] Furniss M.M., Solheim H., Christiansen E. (1990). Transmission of blue-stain fungi by *Ips typographus* (Coleoptera: Scolytidae) in Norway spruce. Ann. Entomol. Soc. Am..

[62] Solheim H. (1993). Fungi associated with the spruce bark beetle *Ips typographus* in an endemic area in Norway. Scan. J. Forest Res..

[63] Viiri H., Weissenberg K. (1995). *Ophiostoma* blue-staining fungi associated with *Ips typographus* in Finland. Akt. Skogforsk..

[64] Ahtiainen J. (2008). The Blue-Stain Fungi Associated with the Spruce Bark Beetle (*Ips typographus* L.) in Lake Vodla Area in Eastern Russia. M.Sc. Thesis.

[65] Solheim H. (1992). The early stages of fungal invasion in Norway spruce infested by the bark beetle *Ips typographus*. Can. J. Bot..

[66] Solheim H. (1992). Fungal succession in sapwood of Norway spruce infested by the bark beetle *Ips typographus*. Eur. J. Forest Pathol..

[67] Solheim H., Långström B. (1991). Blue-stain fungi associated with *Tomicus piniperda* in Sweden and preliminary observations on their pathogenicity. Ann. Sci. For..

[68] Krokene P., Solheim H. (1998). Pathogenicity of four blue-stain fungi associated with aggressive and nonaggressive bark beetles. Phytopathology.

[69] Jacobs K., Kirisits T., Wingfield M.J. (2003). Taxonomic re-evaluation of three related species of *Graphium*, based on morphology, ecology and phylogeny. Mycologia.

[70] Mouton M., Wingfield M.J., van Wyk P.W.J. (1994). *Graphium pseudormiticum* sp. nov.: A new hyphomycete with unusual conidiogenesis. Mycol. Res..

[71] Kirschner R. (1998). Diversität mit Borkenkäfern assoziierter filamentöser Mikropilze. Ph.D. Dissertation.

[72] Plattner A., Kim J.J., Reid J., Hausner G., Lim Y.W., Yamaoka Y., Breuil C. (2009). Resolving taxonomic and phylogenetic incongruence within species *Ceratocystiopsis minuta*. Mycologia.

[73] Bakshi B.K. (1951). Studies on four species of *Ceratocystis*, with a discussion on fungi causing sap-stain in Britain. Mycological Papers.

[74] Harrington T.C., Harrington T.C., Cobb F.W. (1988). *Leptographium* species. Leptographium Root Diseases of Conifers.

[75] Jacobs K., Wingfield M.J. (2001). Leptographium Species—Tree Pathogens,Insect Associates,and Agents of Blue-Stain.

[76] Upadhyay H.P. (1981). A Monograph of Ceratocystis and Ceratocystiopsis.

[77] Hausner G., Reid J., Klassen G.R. (1993). On the phylogeny of *Ophiostoma*, *Ceratocystis* s.s., and *Microascus*, and relationships within *Ophiostoma* based on partial ribosomal DNA sequences. Can. J. Bot..

[78] Hausner G., Reid J., Klassen G.R. (2000). On the phylogeny of members of *Ceratocystis* s.s. and *Ophiostoma* that possess different anamorphic states, with emphasis on the anamorph genus *Leptographium*, based on partial ribosomal DNA sequences. Can. J. Bot..

[79] Jacobs K., Wingfield M.J., Coetsee C., Kirisits T., Wingfield B.D. (2001). *Leptographium guttulatum* sp. nov., a new species from spruce an pine in Europe. Mycologia.

[80] Bakshi B.K. (1950). Fungi associated with ambrosia beetles in Great Britain. Trans. Br. Mycol. Soc..

[81] Zhou X.D., de Beer Z.W., Wingfield B.D., Wingfield M.J. (2002). Infection sequence and pathogenicity of *Ophiostoma ips*, *Leptographium serpens* and *L. lundbergii* to pines in South Africa. Fungal Div..

[82] Zhou X.D., de Beer Z.W., Wingfield B.J., Wingfield M.J. (2002). Infection sequence and pathogenicity of *Ophiostoma ips*, *Leptographium serpens* and *L. lundbergii* to pines in South Africa. Fungal Div..

[83] Solheim H., Krokene P., Långström B. (2001). Effects of growth and virulence of associated blue-stain fungi on host colonization behaviour of the pine shoot beetles *Tomicus minor* and *T. piniperda*. Plant Pathol..

[84] Jacobs K., Bergdahl D.R., Wingfield M.J., Halik S., Seifert K.A., Bright D.E., Wingfield B.D. (2004). *Leptographium wingfieldii* introduced into North America and found associated with exotic *Tomicus piniperda* and native bark beetles. Mycol. Res..

[85] Harding S. (1989). The Influence of Mutualistic Blue-Stain Fungi on Bark Beetle Population Dynamics. Ph.D. Dissertation.

[86] Harrington T.C., McNew D., Steimel J., Hofstra D., Farrell R. (2001). Phylogeny and taxonomy of the *Ophiostoma piceae*-complex and the Dutch elm disease. Mycol. Res..

[87] de Beer Z.W., Wingfield B.D., Wingfield M.J. (2003). The *Ophiostoma piceae* complex in the Southern Hemisphere: A phylogenetic study. Mycol. Res..

[88] Linnakoski R., de Beer Z.W., Rousi M., Niemelä P., Pappinen A., Wingfield M.J. (2008). Fungi, including *Ophiostoma karelicum* sp. nov., associated with *Scolytus ratzeburgi* infesting birch in Finland and Russia. Mycol. Res..

[89] Linnakoski R., de Beer Z.W., Rousi M., Solheim H., Wingfield M.J. (2009). *Ophiostoma denticiliatum* sp. nov. and other *Ophiostoma* species associated with the birch bark beetle in southern Norway. Persoonia.

[90] Masuya H., Kaneko S., Yamaoka Y. (2003). Comparative virulence of blue-stain fungi isolated from Japanese red pine. J. For. Res..

[91] Gorton C., Kim S.H., Henricot B., Webber J., Breuil C. (2004). Phylogenetic analysis of the bluestain fungus *Ophiostoma minus* based on partial ITS rDNA and beta-tubulin sequences. Mycol. Res..

[92] Farrel R.L., Blanchett R.A., Brush T.S., Hadar Y., Iverson S., Krisa K., Wendler P.A., Zimmerman W. (1993). Cartapip™: A biopulping product for control of pitch and resin acid problems in pulp mills. J. Biotechnol..

[93] Zanzot J.W., de Beer Z.W., Eckhardt L.G., Wingfield M.J. (2010). A new *Ophiostoma* species from loblolly pine roots in the southeastern United States. Mycol. Prog..

[94] Jankowiak R., Kolarík M. (2010). Diversity and pathogenicity of ophiostomatoid fungi associated with *Tetropium* species colonizing *Picea abies* in Poland. Folia Microbiol. (Praha).

[95] Paciura D., Zhou X.D., de Beer Z.W., Jacobs K., Wingfield M.J. (2010). Characterization of synnematous bark beetle-associated fungi from China, including *Graphium carbonarium* sp. nov. Fungal Div..

[96] Six D.L., Bourtzis K., Miller T.A. (2003). Bark beetle-fungus symbioses. Insect Symbiosis. Contemporary Topics in Entomology.

[97] Gooday G.W. (1990). Physiology of microbial degradation of chitin and chitosan. Biodegradation.

[98] Asiegbu F.O., Adomas A., Stenlid J. (2005). Conifer root and butt rot caused by *Heterobasidion annosum* (Fr.) Bref. s.l. Mol. Plant. Pathol..

[99] Kirschner R. (2001). Diversity of filamentous fungi in bark beetle galleries in central Europe. Trichomyces and other fungal groups.

[100] Kirisits T. (2010). Fungi isolated from *Picea abies* infested by the bark beetle *Ips typographus* in the Bielowieza forest in north-eastern Poland. Forest Pathol..

[101] Solheim H., Wingfield M.J., Seifert K.A., Webber J.F. (1993). Ecological aspects of fungi associated with *Ips typographus* in Norway. Ceratocystis and Ophiostoma: Taxonomy, Ecology and Pathogenicity.

[102] Six D.L., Bentz B.J. (2007). Temperature determines symbiont abundance in a multipartite bark beetle-fungus ectosymbiosis. Microb. Ecol..

[103] Rice A.V., Thormann M.N., Langor D.W. (2008). Mountain pine beetle-associated blue-stain fungi are differently adapted to boreal temperatures. Forest Pathol..

[104] Bridges J.R., Moser C. (1986). Relationship of phoretic mites (Acari: Tarsonemidae) to the bluestain fungus *Ceratocystis minor*, in trees infested by southern pine beetle (Coleoptera: Scolytida). Environ. Entomol..

[105] Harrington T.C. (1981). Cycloheximide sensitivity as a taxonomic character in *Ceratocystis*. Mycologia.

[106] Hausner G., Reid J., Klassen G.R. (1993). *Ceratocystiopsis*: A reappraisal based on molecular criteria. Mycol. Res..

[107] Hawksworth D.L., Rossman A.Y. (1997). Where are all the undescribed fungi?. Phytopathology.

[108] Grobbelaar J., Aghayeva D., de Beer Z.W., Bloomer P., Wingfield M.J., Wingfield B. (2009). Delimitation of *Ophiostoma quercus* and its synonyms using multiple gene phylogenies. Mycol. Prog..

[109] Bonan G.B., Shugart H. (1989). Environmental factors and ecological processes in boreal forests. Annu. Rev. Ecol. Syst..

[110] Peltonen M., Heliövaara K., Väisänen R. (1997). Forest insects and environmental variation in stand edges. Silva Fenn..

[111] Martikainen P., Siitonen J., Kaila L., Punttila P., Rauh J. (1999). Bark beetles (Coleoptera, Scolytidae) and associated beetle species in mature old-growth boreal forests in southern Finland. For. Ecol. Manage..

[112] Taylor S.W., Carroll A.L., Alfaro R.I., Safranyik L., Safranyik L., Wilson B. (2006). The mountain pine beetle: A synthesis of biology. Management and Impacts in Lodgepole Pine.

[113] Brasier C.M., Mehrotra M.D. (1995). *Ophiostoma himal-ulmi* sp. nov., a new species of Dutch elm disease fungus endemic to the Himalayas. Mycol. Res..

[114] Heybroek H.M., Stipes R.J., Campana R.J. (1981). Elm cultivation. Compendium of Elm Diseases.

[115] Ozolin G.P., Kryukova E.A., Kargov (1980). Methods of controlling Dutch elm disease by selection, breeding and introduction. The Enhancement of the Stability and Longevity of Protective Forest Plantations.

[116] Mattson W., Vanhanen H., Veteli T., Sivonen S., Niemelä P. (2007). Few immigrant phytophagous insects on woody plants in Europe: Legacy of the European crucible?. Biol. Invasions.

[117] Karjalainen T., Toppinen A. (2007). Russia raising export tariffs. EFI News.

[118] Ayres M.P., Lombardero M.J. (2000). Assessing the consequences of global change for forest disturbance from herbivores and pathogens. Sci. Total Environ..

[119] Jönsson A.M., Harding S., Bärring L., Ravn H.P. (2007). Impact of climate change on the population dynamics of Ips typographus in southern Sweden. Agr. Forest Meteorol..

[120] Pouttu A., Annila E. (2010). Kirjanpainajalla kaksi sukupolvea kesällä 2010. Metsätieteen aikakauskirja.

[121] Långström B., Lindelöw Å., Schroeder L.M., Björklund N., Öhrn P. The spruce bark beetle outbreak in Sweden following the January-storms in 2005 and 2007. Insects and Fungi in Storm Areas, Proceedings of Workshop of IUFRO Working Party 7.03.10.

